# Metformin as adjuvant therapy in obese knee osteoarthritis patients

**DOI:** 10.1007/s10787-024-01495-y

**Published:** 2024-06-13

**Authors:** Amany Abd Elaal Aiad, Sahar Mohamed El-Haggar, Amal Mohamed El-Barbary, Dalia Refat El-Afify

**Affiliations:** 1 Department of Clinical Pharmacy, Faculty of Pharmacy, Tanta University, Tanta, 31527 Egypt; 2 Department of Physical Medicine, Rheumatology and Rehabilitation, Faculty of Medicine, Tanta University, Tanta, 31527 Egypt

**Keywords:** Knee osteoarthritis, Metformin, COMP, CTX-1, IL-1β, WOMAC

## Abstract

**Aims:**

This study aimed at investigating the efficacy of metformin as adjuvant therapy for obese knee osteoarthritis (OA) patients, considering its anti-inflammatory and cartilage-protective effects.

**Patients and methods:**

In this randomized, double-blind, placebo-controlled study, 50 obese knee OA patients were assigned randomly to two groups, the metformin group (*n* = 25) which was treated with metformin 500 mg orally BID plus celecoxib 200 mg orally once daily, and the placebo group (*n* = 25) which was treated with placebo tablets BID plus celecoxib 200 mg orally once daily for 12 weeks. Cartilage Oligomeric Matrix Protein (COMP), C-terminal cross-linked telopeptide of type I collagen (CTX-1), and Interleukin 1-beta (IL-1β) serum levels were measured, while Western Ontario and McMaster Universities Arthritis Index (WOMAC) score assessed knee pain, stiffness, and physical function at baseline and after 12 weeks.

**Results:**

Following a 12-week treatment, the metformin group exhibited significantly reduced levels of COMP, CTX-1, and IL-1β in the serum compared to the placebo group (*p* = 0.0081, *p* = 0.0106, and *p* = 0.0223, respectively). Furthermore, metformin group produced significant improvements in WOMAC total scale (*p* < 0.0001), specifically in knee pain, stiffness, and physical function compared to placebo group (*p* < 0.0001, *p* < 0.0001, and *p* < 0.0001, respectively).

**Conclusion:**

Metformin as an adjuvant therapy in obese knee OA patients may have beneficial effects on cartilage degradation and inflammation, as evidenced by the significant decreases in serum COMP, CTX-1, and IL-1β levels. Additionally, metformin may improve clinical outcomes, as shown by the significant improvements in WOMAC scores.

**Clinicaltrials.gov ID:**

NCT05638893/Registered December 6, 2022 — Retrospectively.

## Introduction

Osteoarthritis (OA) is a chronic joint disease that is highly prevalent worldwide, with the knee joint being the most commonly affected, it causes joint pain and physical disability, leading to a decrease in mobility and quality of life (Bierma-Zeinstra 2019; Quicke et al. 2022). OA is characterized by cartilage degeneration and low-grade inflammation (Yao et al. 2023; Allen et al. 2022). It affects the entire joint, resulting in cartilage loss, remodeling of subchondral bone, and osteophyte formation. The role of obesity and metabolic syndrome in pathogenesis of OA was previously reported (Puenpatom and Victor 2009).

The increased mechanical load on joints in obese individuals has been linked to knee OA. However, obesity is linked to hand osteoarthritis, indicating potential systemic involvement. With the global increase in the elderly population and the prevalence of obesity, there is an anticipated rise in the incidence of osteoarthritis, necessitating the identification of effective drugs that can modify its progression (Lambova et al. 2023; Gamze et al. 2023).

Currently, there are no approved disease-modifying drugs for osteoarthritis, and the management mainly depends on symptomatic treatment. End-stage osteoarthritis is usually treated by joint replacement surgery to enhance function and quality of life (Lambova 2023a). Celecoxib, a non-steroidal anti-inflammatory drug (NSAID), is commonly used to manage osteoarthritis symptoms, but its long-term use is associated with adverse effects such as gastrointestinal complications and cardiovascular events (Richard et al. 2023). A major challenge in developing therapeutic strategies for osteoarthritis is not only to alleviate pain, but also to have the potential to stop or prevent articular cartilage degeneration and subchondral bone changes (Lambova et al. 2023).

Metformin, a safe and well-tolerated oral medication prescribed as a first-line treatment for obese type-2 diabetic patients, has shown potential in slowing down the progression of OA (Ziqubu et al. 2023). Previous in vitro and animal studies reported that metformin may exert beneficial effects in OA through various mechanisms, including its anti-inflammatory properties, antioxidant effects, modulation of the microbiome and upregulation of autophagy (Lambova 2023b; Lim et al. 2022; Feng et al. 2020; Wang et al. 2018, 2020; Belenska-Todorova et al. 2021; He et al. 2020; Li et al. 2020a, b; Na et al. 2021). Furthermore, metformin has been suggested to have a suppressive effect on osteoclastogenesis and reduction in Receptor activator of nuclear factor kappa-b ligand (RANKL) expression in osteoblasts (Mai et al. 2011). Previous retrospective studies found that patients with diabetes type 2 and OA who were treated with metformin and cyclooxygenase-2 (COX-2) inhibitors experienced a decreased occurrence of joint replacement compared to patients who received only COX-2 inhibitors (Lu et al. 2018; Lai et al. 2022). Additionally, a prospective cohort study showed that individuals with obesity and knee OA who used metformin had a decreased medial cartilage volume loss rate and a tendency towards a lower risk of total knee replacement (Wang et al. 2019). Evidence from animal and epidemiological studies has indicated that metformin could be a beneficial treatment approach for knee OA management. However, there is currently a lack of high-quality clinical trials that support this notion.

This clinical study designed to investigate the efficacy of metformin as adjuvant therapy in obese knee OA patients, using the western ontario and McMaster universities arthritis index (WOMAC) score (McConnell et al. 2001) and by evaluating serum levels of cartilage oligomeric matrix protein (COMP), C-terminal cross-linked telopeptide of type I collagen (CTX-1), and Interleukin 1-beta (IL-1β).

## Patients and methods

### Study design

In this prospective, randomized, double-blind, placebo-controlled study, obese patients with knee OA were enrolled from the outpatient clinic of the physical medicine and rheumatology department at Tanta university hospital in Tanta, Egypt. The study was carried out in accordance with the ethical standards of Helsinki Declaration in 1964 and its later amendments. The study was approved by the ethical standards of Tanta University Research Ethical Committee (approval code: 34,363/1/21). The study was registered as clinical trial at clinicaltrial.gov with ID: NCT05638893 under the title: metformin as adjuvant therapy in obese knee osteoarthritis patients.

### Patients

A total number of 50 patients were randomized into two groups; the placebo group (*n* = 25) and the metformin group (*n* = 25). Participants were recruited between Feb 1, 2021, and Mar 12, 2022. Inclusion criteria were patients with obesity (BMI ≥ 30 kg/m^2^) of both sex, age ≥ 45 years old, and patients with symptomatic and radiological evidence of OA in one or both knee joints according to European League Against Rheumatism (EULAR) guidelines (Zhang et al. 2010). The exclusion criteria were patients with other rheumatic diseases, infection-induced osteoarthritis, crystal deposition arthritis, patients with chronic diseases (including diabetes mellitus, hypertension, renal or hepatic impairment), positive malignancy, active peptic or duodenal ulcer and those who had received an intra-articular steroid injection, pregnancy or lactation. All participants provided written informed consent prior to their involvement in the study.

### Randomization and masking

Patients were randomized to either the metformin or placebo group in a 1:1 ratio using simple randomization by computer-generated random numbers and allocation concealment was carried out using sealed envelopes. All participants, investigators, outcome assessors, and statisticians were unaware of the group allocation.

### Procedures

The patients were randomized into two groups; the placebo group (*n* = 25) which received placebo tablet twice daily plus celecoxib (celeborg^®^; Borg Pharmaceutical Industries, Alexandria, Egypt) 200 mg capsule once daily orally for 12 weeks, and the metformin group (*n* = 25) which received metformin (Cidophage^®^; CID Co., Cairo, Egypt) 500 mg tablet orally twice daily plus celecoxib (celeborg^®^) 200 mg capsule orally once daily for 12 weeks. Patients who were taking NSAIDs were advised to stop their medication for at least 2 weeks before the initial dose of celecoxib. All patients adhered to a standardized balanced diet, carefully evaluated by a nutritionist to minimize the impact of diet on weight loss. Furthermore, patients were advised to maintain their usual level of physical activity consistently throughout the study period.

### Demographic, anthropometric and clinical data

Data on age, gender, smoking, disease duration, number of symptomatic knees, and Kellgren and Lawrence grading scale (KL) were collected. Anthropometric measurements, including weight and height, were taken to calculate body mass index [BMI = Weight (kg)/Height (m^2^)] at baseline and 12 weeks after the assigned treatment.

### Assessment of knee symptoms

Knee symptoms were assessed by Western Ontario and McMaster Universities Arthritis Index (WOMAC) score at baseline and 12 weeks after treatment. The evaluation involved the comprehensive assessment of the overall index and specific subscales, including joint pain (consisting of 5 questions), stiffness (comprising 2 questions), and physical function limitation (comprising 17 questions). Each question was assigned a rating on a scale of 0 to 4, where 0 indicated the absence of symptoms and 4 indicated the presence of severe symptoms. Consequently, the highest possible scores for pain, stiffness, physical function limitation, and the total index were 20, 8, 68, and 96 points, respectively.

### Biochemical measurements

At baseline and after 12 weeks of treatment, each participant provided a 3 ml venous blood sample after overnight fasting through antecubital venipuncture between 8:30 and 10:30 am. The blood was collected in a plain test tube and subsequently centrifuged at 3000 rpm for 20 min (Hettich Zentrifugen EPA 20). The resulting serum samples were stored at − 80 °C until biochemical analysis. Cartilage Oligomeric Matrix Protein (COMP), C-terminal cross-linked telopeptide of type I collagen (CTX-1), and Interleukin 1-beta (IL-1β) were assayed through the double-antibody sandwich technique using the commercially available enzyme-linked immunosorbent assay (ELISA) kits (SunRed Biotechnology Co., China, Catalogue No. 201-12-1487B, 201-12-1350D, and 201-12-0144, respectively). Following the instructions provided by the manufacturer, the assays for these biomarkers were conducted.

### Assessment of patients’ adherence and drugs tolerability

Patients were followed up by weekly telephone calls and face to face meetings every 2 weeks in order to supply the participants with medications, and report any potential adverse effects. Participant adherence was evaluated based on the rate of medication refills. Drug-related toxicity was assessed according to common terminology criteria for adverse events version 5.0 (CTCAE version 5.0).

### Primary and secondary outcomes

The primary outcome was the change in WOMAC score, which was evaluated in both groups before and after the treatment. The secondary outcomes were the change in serum levels of biological biomarkers including COMP, CTX-1, and IL-1β over 12 weeks of treatment.

### Sample size calculation

No previous clinical studies were available to estimate the effect of metformin on change of WOMAC total score in patients with knee osteoarthritis. Based on Dougados et al. (2000) who reported that clinical significant response defined as 20% improvement in WOMAC total score, a sample size of 22 in each group would provide an 80% statistical power to identify the potential beneficial effect of metformin on WOMAC total score when compared to the control group assuming a mean difference 20% between the two groups in WOMAC total score, α-error of 0.05, and β-error of 0.2. Assuming that, the attrition rate is 10%, the sample size would be 25 patients in each group.

### Statistical analysis

The statistical analysis was performed using the SPSS statistical package version 26.0 (April 2019) developed by IBM corporation’s software group in the United States. Quantitative variables were presented as mean ± standard deviation (SD), while qualitative variables were represented as percentages (%). Normality test was done by Shapiro–Wilk test. For normally distributed data, we used unpaired student *t* test to compare the mean values between the two groups, and paired student *t* test to compare mean values within the same group before and after treatment. For non-normally distributed data, we used Mann–Whitney test to compare the mean values between the two groups, and Wilcoxon matched-pairs signed rank test to compare mean values within the same group before and after treatment. To compare categorical data between the two groups, the Chi Square analysis was employed. Fisher’s exact test was used for categorical data to examine adverse effects reported. Spearman correlation analysis was employed to examine correlations between the serum concentrations of COMP, CTX-1, IL-1β, and WOMAC score. All statistical tests were two-tailed, and a significance level of *p* < 0.05 was considered statistically significant.

## Results

**Fig.1:**
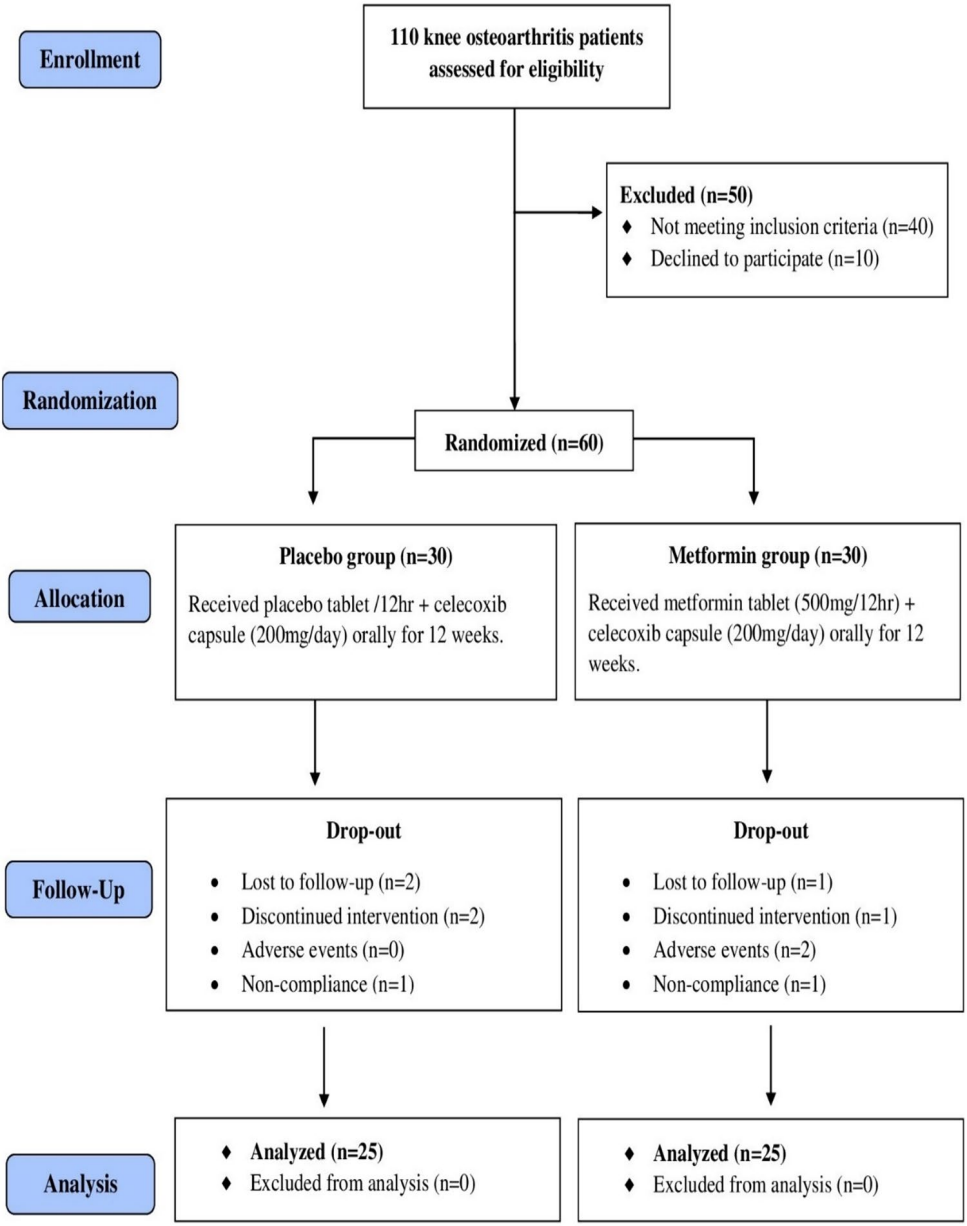
CONSORT Flow Diagram of the study participants

Among the 110 patients evaluated for eligibility with knee OA, 50 patients were excluded (40 patients who did not meet the inclusion criteria and 10 patients who declined to participate). Consequently, a total of 60 patients were randomly assigned to the two study groups, comprising 30 patients in the placebo group and 30 patients in the metformin group. Throughout the study, ten patients from both study groups were dropped-out due to reasons such as lost to follow-up, discontinued intervention, adverse events, non-compliance, and the preliminary data of those patients were excluded from the final analysis. In this context, the final analysis consisted of a total of 50 patients (25 participants in each group). CONSORT flow diagram of the study participants is shown in Fig. 1.

### Patients’ characteristics

Regarding demography, anthropometric, and clinical data, there was statistically non-significant difference between the two groups concerning age, gender, smoking, weight, height, BMI, disease duration, number of symptomatic knees, KL grading, WOMAC total scale, and its three subscales at baseline (*p*˃0.05) as postulated in Table 1**.**

**Table 1:** Baseline demographic, anthropometric, and clinical data of the study participants

	Placebo group (*n* = 25)	Metformin group (*n* = 25)	*p*-value
Demographic data			
Age (years)	52.28 ± 6.100	51.00 ± 5.752	0.4859
Gender			
Male	3 (12%)	3 (12%)	1
Female	22 (88%)	22 (88%)	–
Smoking			
Smoker	1 (4%)	1 (4%)	1
Non-smoker	24 (96%)	24 (96%)	–
Anthropometric data			
Weight (kg)	98.84 ± 16.38	96.08 ± 14.79	0.6200
Height (cm)	160.2 ± 6.444	162.6 ± 6.868	0.2241
BMI (kg/m^2^)	38.49 ± 5.934	36.40 ± 5.555	0.1713
Clinical data			
Disease duration (Years)			
Below 5	10 (40%)	9 (36%)	0.9373
From 6 to 10	9 (36%)	9 (36%)	–
Over 10	6 (24%)	7 (28%)	–
Number of symptomatic Knees			
Right	4 (16%)	5 (20%)	0.8406
Left	4 (16%)	5 (20%)	–
Bilateral	17 (68%)	15 (60%)	–
KL score	2.48 ± 0.6532	2.44 ± 0.6506	0.8905
WOMAC total scale	61.68 ± 6.625	61.08 ± 7.359	0.7632
WOMAC pain subscale	11.96 ± 1.695	12.00 ± 2.062	0.7877
WOMAC stiffness subscale	5.68 ± 0.8021	5.92 ± 0.9539	0.4184
WOMAC function subscale	44.04 ± 4.695	43.16 ± 5.406	0.5418
COMP (ng/mL)	1370 ± 727.7	1248 ± 679.5	0.5035
CTX-1 (ng/mL)	12.99 ± 3.583	12.01 ± 4.018	0.4325
IL-1β (pg/mL)	2537 ± 785.2	2384 ± 821.2	0.3753

Data are presented as mean ± SD, number and percentage

*BMI* body mass index; *KL score* Kellgren and Lawrence grading scale, *WOMAC* western ontario and McMaster universities arthritis index; *COMP* cartilage oligomeric matrix protein, *CTX-1* C-terminal cross-linked telopeptide of type I collagen; *IL-1β* interleukin 1-beta.

### Effect of intervention on body weight and BMI

For metformin group, mean body weight and mean BMI was significantly lower after treatment than its mean value at baseline (*p* < 0.0001 and *p* < 0.0001, respectively). Following a 12-week treatment period, the metformin group exhibited a lower mean body weight and a significantly lower mean BMI compared to the placebo group (*p* = 0.0981 and *p* = 0.0106, respectively). Changes in body weight and BMI at baseline and after 12 weeks are illustrated in Table 2.

**Table 2:** Markers in the two study groups at baseline and after 12 weeks of treatment

	Placebo group (*n* = 25)	Metformin group (*n* = 25)	^b^*p*-value
Body weight (kg)			
At baseline	98.84 ± 16.38	96.08 ± 14.79	0.6200
After 12 weeks	99.24 ± 16.49	91.68 ± 14.78	0.0981
^a^*p*-value	0.3385	< 0.0001*	–
BMI (kg/m^2^)			
At baseline	38.49 ± 5.934	36.40 ± 5.555	0.1713
After 12 weeks	38.66 ± 6.066	34.72 ± 5.502	0.0106*
^a^*p*-value	0.1864	< 0.0001*	–
WOMAC total scale (0–96)			
At baseline	61.68 ± 6.625	61.08 ± 7.359	0.7632
After 12 weeks	54.32 ± 9.072	40.44 ± 6.564	< 0.0001*
^a^*p*-value	0.0002*	< 0.0001*	–
WOMAC pain subscale (0–20)			
At baseline	11.96 ± 1.695	12.00 ± 2.062	0.7877
After 12 weeks	10.24 ± 1.877	7.68 ± 1.406	< 0.0001*
^a^*p*-value	< 0.0001*	< 0.0001*	–
WOMAC stiffness subscale (0–8)			
At baseline	5.68 ± 0.8021	5.92 ± 0.9539	0.4184
After 12 weeks	4.96 ± 0.9345	3.72 ± 0.7916	< 0.0001*
^a^*p*-value	< 0.0001*	< 0.0001*	–
WOMAC function subscale (0–68)			
At baseline	44.04 ± 4.695	43.16 ± 5.406	0.5418
After 12 weeks	39.12 ± 7.270	29.04 ± 4.979	< 0.0001*
^a^*p*-value	0.0034*	< 0.0001*	–
COMP (ng/mL)			
At baseline	1370 ± 727.7	1248 ± 679.5	0.5035
After 12 weeks	1314 ± 666.8	903.6 ± 585.4	0.0081*
^a^*p*-value	0.1645	< 0.0001*	–
CTX-1 (ng/mL)			
At baseline	12.99 ± 3.583	12.01 ± 4.018	0.4325
After 12 weeks	12.90 ± 3.574	10.47 ± 4.080	0.0106*
^a^*p*-value	0.6150	< 0.0001*	–
IL-1β (pg/mL)			
At baseline	2537 ± 785.2	2384 ± 821.2	0.3753
After 12 weeks	2399 ± 789.4	1920 ± 776.1	0.0223*
^a^*p*-value	0.0219*	< 0.0001*	–

Data are presented as mean ± SD

*BMI* body mass index; *WOMAC* western ontario and McMaster universities arthritis index; *COMP* cartilage oligomeric matrix protein; *CTX-1* C-terminal cross-linked telopeptide of type I collagen; *IL-1β* interleukin 1-beta.

^a^*p-*value before versus after treatment within the same group; ^b^*p-*value *p*-value between the two groups after treatment, *statistically-significant difference (*p* < 0.05).

### Effect of intervention on WOMAC score

For metformin group, the mean values for WOMAC total scale, WOMAC pain subscale, WOMAC stiffness subscale, and WOMAC function subscale were significantly lower after treatment compared to their respective mean values at baseline (*p* < 0.0001, *p* < 0.0001, *p* < 0.0001, and *p* < 0.0001, respectively). Following a 12-week treatment period, the metformin group exhibited significantly lower mean values for WOMAC total scale and its subscales in comparison with the placebo group (*p* < 0.0001, *p* < 0.0001, *p* < 0.0001, and *p* < 0.0001, respectively). Changes in WOMAC scores at baseline and after 12 weeks are presented in Table 2.

### Effect of intervention on the measured biological markers

At baseline, non-statistically significant differences were observed in COMP, CTX-1, and IL-1β serum levels between the two study groups (*p* > 0.05). However, in the metformin group, the mean values of COMP, CTX-1, and IL-1β were significantly lower after treatment compared to their respective mean values at baseline (*p* < 0.0001, *p* < 0.0001, and *p* < 0.0001*,* respectively). Following a 12-week treatment period, the mean values of COMP, CTX-1, and IL-1β were significantly lower in the metformin group compared to the placebo group (*p* = 0.0081, *p* = 0.0106, and *p* = 0.0223*,* respectively). Changes in COMP, CTX-1, and IL-1β serum levels at baseline and after 12 weeks are presented in Table 2.

### Correlation analysis

The correlations between WOMAC score and the serum level of each of COMP, CTX-1, and IL-1β were performed for metformin group at baseline and after the treatment. The serum level of COMP, CTX-1, and IL-1β showed a statistically significant positive correlation with WOMAC total scale at baseline (*p* < 0.001, *p* = 0.008, and *p* = 0.002, respectively), and also the serum level of COMP, CTX-1, and IL-1β showed a statistically significant positive correlation with WOMAC total scale after the treatment (*p* < 0.001, *p* < 0.001, and *p* < 0.001, respectively). Alternatively, the serum level of COMP, CTX-1, IL-1β, and WOMAC total scale showed a statistically significant negative correlation with % weight loss after the treatment (*p* < 0.001, *p* = 0.005, *p* = 0.004, and *p* < 0.001, respectively).

**Fig.2:**
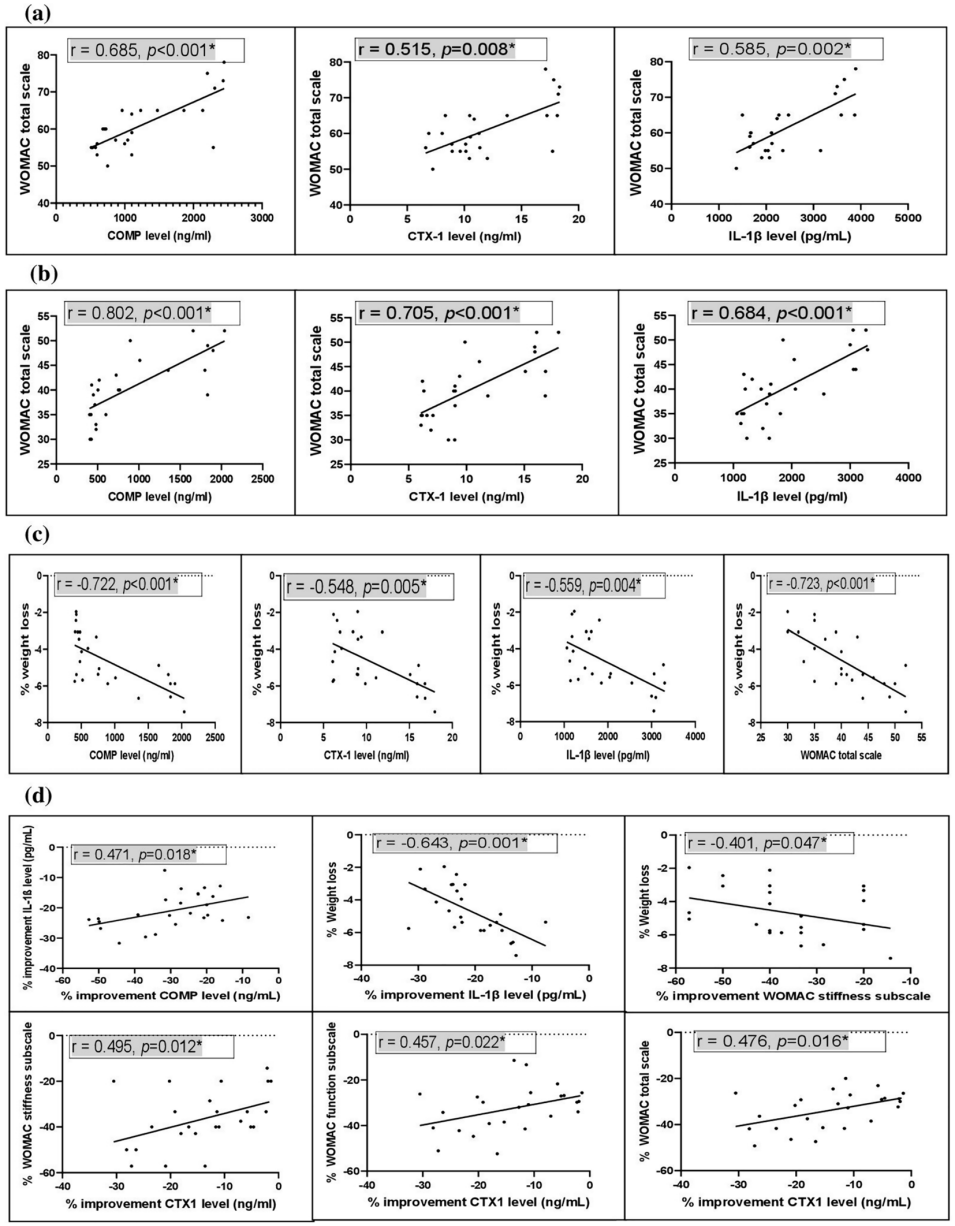
Correlation analysis. Correlation between WOMAC total scale and the serum level of each of COMP, CTX-1, and IL-1β for metformin group (**a**) at baseline and (**b**) after the treatment. (**c**) Correlation between % weight loss and the serum level of each of COMP, CTX-1, IL-1β, and WOMAC total scale for metformin group after the treatment. (**d**) Correlation between % improvement in COMP level with % improvement in IL-1β level. Correlation between % improvement in IL-1β level and % improvement in WOMAC stiffness subscale with % weight loss. Correlation between % improvement in CTX1 level with % improvement in WOMAC stiffness subscale, % improvement in WOMAC function subscale, and % improvement in WOMAC total scale after treatment in metformin group.*Significant difference (*p* < 0.05)

For additional analysis of the data, the correlations between % improvement in WOMAC score and % improvement in the serum level of each of COMP, CTX-1, and IL-1β were performed for metformin group. It was found that % improvement in the serum level of COMP has statistically significant positive correlation with % improvement in the serum level of IL-1β (*p* = 0.018), and also % improvement in the serum level of CTX-1 has statistically significant positive correlation with % improvement in WOMAC stiffness subscale, % improvement in WOMAC function subscale, and % improvement in WOMAC total scale (*p* = 0.012, *p* = 0.022, and *p* = 0.016, respectively). Alternatively, % improvement in IL-1β serum level and % improvement in WOMAC stiffness subscale exhibited a statistically significant negative correlation with % weight loss (*p* = 0.001 and *p* = 0.047, respectively) as shown in Fig. 2.

### Analysis of side effects and drug tolerability

No instances of lactic acidosis or hypoglycemia were observed as adverse effects among the patients. Only gastrointestinal tract-related side effects were reported, that appeared in the beginning of taking medication and subsided over time. Non-statistically significant differences were observed between the two study groups in relation to the reported side effects (*p* > 0.05). Abdominal pain was reported in both groups. Diarrhea and nausea only reported in metformin group. In both groups, all reported side effects were categorized as grade 1 and 2 in accordance with the Common Terminology Criteria for Adverse Events version 5.0 (CTCAE version 5.0), and no severe adverse effects were observed in either group. The reported side effects are illustrated in Table 3.

**Table 3:** The reported side effects in the two studied groups

Side effects		Placebo group (*n* = 25)	Metformin group (*n* = 25)	^a^*p*-value
GIT symptoms				
Abdominal pain	All grades (total)	3 (12%)	5 (20%)	0.7019
	Grade 1	2 (8%)	2 (8%)	
	Grade 2	1 (4%)	3 (12%)	
Diarrhea	All grades (total)	0 (0%)	3 (12%)	0.2347
	Grade 1	0 (0%)	2 (8%)	
	Grade 2	0 (0%)	1 (4%)	
Nausea	All grades (total)	0 (0%)	1 (4%)	> 0.999
	Grade 1	0 (0%)	1 (4%)	
	Grade 2	0 (0%)	0 (0%)	

Data are presented as number and percentage (%) according to common terminology criteria for adverse events version 5.0 (CTCAE version 5.0)

*GIT* gastrointestinal tract

^a^Data were analyzed by Fisher’s exact test

## Discussion

Obesity and chronic low-grade inflammation are risk factors for developing OA (Lambova et al. 2023). Metformin is antidiabetic drug that has been shown to have anti-inflammatory and chondroprotective effects in preclinical studies (Lim et al. 2022), and it also helps in weight-loss in non-diabetic individuals through improving insulin sensitivity (Hui et al. 2019). Our research aimed to investigate the efficacy of metformin as adjuvant therapy in obese knee OA patients.

The findings of our study demonstrated that the metformin group reduced body weight and BMI in comparison with the placebo group after treatment, without causing hypoglycemia as a side effect (Lambova 2023b), and this is compatible with the results of other previous studies (Hui et al. 2019; Ning et al. 2018; Abed et al. 2014). Metformin promotes weight loss through improving insulin resistance, decreasing gastrointestinal absorption of carbohydrates, it also has lipolytic and anorectic effect through its ability to modify the appetite regulatory centers in the hypothalamus and it can decrease leptin levels (Hui et al. 2019; Yerevanian and Soukas 2019).

The findings from our study revealed a significant reduction in WOMAC score, COMP, CTX-1, and IL-1β levels in the metformin group when compared to the placebo group.

The WOMAC score is a widely used clinical marker to assess the severity of OA symptoms, including pain, stiffness, and physical function, the effect of metformin on WOMAC score was not previously studied. In our study, the use of metformin as adjuvant therapy with celecoxib produced a statistically significant decrease in WOMAC score when compared with celecoxib and placebo, and the decrease in total WOMAC had a significant correlation with weight loss, which is similar to previous studies that reported direct correlation between weight loss and the reduction of pain as well as improvement in clinical function in osteoarthritis (Panunzi et al. 2021). The ability of metformin to reduce pain in OA patients as also reported by others, was explained by metformin induced activation of AMP-activated protein kinase (AMPK), and AMPK activation inhibits mammalian target of rapamycin complex 1 (mTORC1) that participates in pain transmission (Song et al. 2022; Baeza-Flores et al. 2020; Xiang et al. 2019; Gregory et al. 2010). Moreover, the significant decrease in the proinflammatory cytokine IL-1β in metformin group may also explain reducing inflammatory pain (Xiang et al. 2019).

Articular cartilage is comprised of chondrocytes and extracellular matrix (ECM) consisting of proteoglycans and collagens. COMP, a marker of cartilage degradation, plays a vital role in collagen assembly and the maintenance of ECM stability (Lambova et al. 2023), and may predict OA severity (Hao et al. 2019). In our study, metformin group had a significant decrease in COMP levels compared to placebo group, which suggests that metformin may produce chondroprotective effects in OA as previously described by in vitro studies, and may be due to enhancement of autophagy and resetting the balance between autophagy and apoptosis in chondrocytes through activation of AMPK and inducing the silent mating type information regulation 1 (SIRT1) expression (Wang et al. 2020). In addition, metformin decreases matrix-degrading enzymes expression in chondrocytes therefore decreasing matrix degradation (Li et al. 2023). In our study, the decrease in COMP was found to be significantly correlated to weight loss, which is consistent with previous findings from other studies (Lambova et al. 2023; Bartels et al. 2014), and this may reflect that weight loss can decrease the mechanical loading on weight-bearing joints and cartilage degradation.

Bone remodeling represents a balance between osteoblast-mediated bone formation and osteoclast-mediated resorption in order to maintain the integrity of bone tissue. The aberrant differentiation of osteoclasts or osteoblasts can result in the microstructural deterioration of subchondral bone, which in turn can contribute to articular cartilage damage (Mai et al. 2011; Li et al. 2022). CTX-1 is a marker of bone resorption. In our study, metformin group had significantly reduced CTX-1 level compared to the placebo group and the decrease in CTX-1 was significantly correlated to weight loss which was also observed in preclinical studies (Li et al. 2022). Metformin can regulate subchondral bone remodeling through enhancement of osteoblast differentiation, decreasing Receptor Activator of Nuclear Factor Kappa-B Ligand (RANKL) expression in osteoblasts and therefore suppression of osteoclastogenesis (Mai et al. 2011; Ma et al. 2018), and this may explain the decrease in CTX-1 level in metformin group.

The development of osteoarthritis is significantly influenced by IL-1β, a pro-inflammatory cytokine that assumes a critical role (Mohammed et al. 2014). Our results showed that the metformin group had significantly reduced IL-1β level than the placebo group which is compatible with the results of other previous studies (Li et al. 2022; Mohammed et al. 2014). Metformin activates AMPK; AMPK activation has been shown to have anti-inflammatory effects by suppressing nuclear factor kappa B (NF-kB) with subsequent inhibition of the overexpression of IL-1β (Xiang et al. 2019; Zhang et al. 2020).

According to our results, we found that metformin may improve clinical outcomes, as shown by the significant improvements in WOMAC scores and it can decrease cartilage degeneration, inflammation and bone resorption.

The current study reported gastrointestinal side effects such as abdominal pain, diarrhea, and nausea. While the occurrence of these side effects was more frequent in the metformin group compared to the placebo group, the difference did not reach statistical significance. Notably, these side effects occurred early in the treatment, were mild and transient in nature, and resolved with continued medication use. Administration of the study medications after a meal helped alleviate these gastrointestinal side effects.

The overall findings from the present study demonstrated the tolerability of metformin and its efficacy as adjuvant therapy to celecoxib in obese patients with knee osteoarthritis through its favorable effects on clinical outcome (WOMAC score) and biological markers involved in osteoarthritis (COMP, CTX-1 and IL-1β) and through reduction in body weight and BMI.

There are limitations in our study include the relatively small sample size of the studied groups, our study population was limited to the residents of Tanta city. Conducting additional studies on diverse ethnic groups and larger sample size are required. In addition, the study employed a small metformin dose of 500 mg twice daily and exploring the efficacy and safety of higher metformin doses on obese knee osteoarthritis patients can be a future research.

## Conclusion

Metformin as an adjuvant therapy in obese knee OA patients may have beneficial effects on cartilage degradation and inflammation, as evidenced by the significant decreases in serum COMP, CTX-1, and IL-1β levels. Additionally, metformin may improve clinical outcomes, as shown by the significant improvements in WOMAC scores. The possible mechanisms of action of metformin in reducing the severity of OA symptoms include its anti-inflammatory and chondroprotective effects, and regulation of bone remodeling and metabolic function. Further studies are needed to confirm these findings and elucidate the exact mechanism of action of metformin in OA.

## Data Availability

Data and analytic methods will be available, upon reasonable request, from the corresponding author, Amany Abd Elaal Aiad, email: amany_43927_pg@pharm.tanta.edu.eg.
